# Composite quantitative knee structure metrics predict the development of accelerated knee osteoarthritis: data from the osteoarthritis initiative

**DOI:** 10.1186/s12891-020-03338-7

**Published:** 2020-05-13

**Authors:** Matthew S. Harkey, Julie E. Davis, Lori Lyn Price, Robert J. Ward, James W. MacKay, Charles B. Eaton, Grace H. Lo, Mary F. Barbe, Ming Zhang, Jincheng Pang, Alina C. Stout, Bing Lu, Timothy E. McAlindon, Jeffrey B. Driban

**Affiliations:** 1 Division of Rheumatology, Allergy, & Immunology, Tufts Medical Center, 800 Washington Street, Box 406, Boston, MA 02111 USA; 2 Department of Population and Quantitative Health Sciences, University of Massachusetts Medical School, Worcester, MA USA; 3 Department of Global Health in the Milken Institute of Public Health, George Washington University, Washington, DC USA; 4 The Institute for Clinical Research and Health Policy Studies, Tufts Medical Center, Boston, MA USA; 5 Tufts Clinical and Translational Science Institute, Tufts University, Boston, MA USA; 6 Department of Radiology, Tufts Medical Center, Boston, MA USA; 7 Norwich Medical School, University of East Anglia, Norwich, UK; 8 Center for Primary Care and Prevention, Alpert Medical School of Brown University, Pawtucket, RI USA; 9 Medical Care Line and Research Care Line, Houston Health Services Research and Development Center of Excellence Michael E. DeBakey VAMC, Houston, TX USA; 10 Section of Immunology, Allergy, and Rheumatology, Baylor College of Medicine, Houston, TX USA; 11 Department of Anatomy and Cell Biology, Temple University School of Medicine, Philadelphia, PA USA; 12 Department of Computer Science & Networking, Wentworth Institute of Technology, Boston, MA USA; 13 Pfizer Inc., Cambridge, MA USA; 14 Public Health Institute, Northeastern University, Boston, MA USA; 15 Division of Rheumatology, Immunology & Allergy, Brigham & Women’s Hospital and Harvard Medical School, Boston, MA USA

**Keywords:** Magnetic resonance imaging, Cartilage, Bone marrow lesions, Effusion, Synovitis

## Abstract

**Background:**

We aimed to determine if composite structural measures of knee osteoarthritis (KOA) progression on magnetic resonance (MR) imaging can predict the radiographic onset of accelerated knee osteoarthritis.

**Methods:**

We used data from a nested case-control study among participants from the Osteoarthritis Initiative without radiographic KOA at baseline. Participants were separated into three groups based on radiographic disease progression over 4 years: 1) accelerated (Kellgren-Lawrence grades [KL] 0/1 to 3/4), 2) typical (increase in KL, excluding accelerated osteoarthritis), or 3) no KOA (no change in KL). We assessed tibiofemoral cartilage damage (four regions: medial/lateral tibia/femur), bone marrow lesion (BML) volume (four regions: medial/lateral tibia/femur), and whole knee effusion-synovitis volume on 3 T MR images with semi-automated programs. We calculated two MR-based composite scores. Cumulative damage was the sum of standardized cartilage damage. Disease activity was the sum of standardized volumes of effusion-synovitis and BMLs. We focused on annual images from 2 years before to 2 years after radiographic onset (or a matched time for those without knee osteoarthritis). To determine between group differences in the composite metrics at all time points, we used generalized linear mixed models with group (3 levels) and time (up to 5 levels). For our prognostic analysis, we used multinomial logistic regression models to determine if one-year worsening in each composite metric change associated with future accelerated knee osteoarthritis (odds ratios [OR] based on units of 1 standard deviation of change).

**Results:**

Prior to disease onset, the accelerated KOA group had greater average disease activity compared to the typical and no KOA groups and this persisted up to 2 years after disease onset. During a pre-radiographic disease period, the odds of developing accelerated KOA were greater in people with worsening disease activity [versus typical KOA OR (95% confidence interval [CI]): 1.58 (1.08 to 2.33); versus no KOA: 2.39 (1.55 to 3.71)] or cumulative damage [versus typical KOA: 1.69 (1.14 to 2.51); versus no KOA: 2.11 (1.41 to 3.16)].

**Conclusions:**

MR-based disease activity and cumulative damage metrics may be prognostic markers to help identify people at risk for accelerated onset and progression of knee osteoarthritis.

## Background

Knee osteoarthritis is typically considered a gradually progressive disease that is a leading cause of physical disability [1]. However, at least 20% of people who develop knee osteoarthritis will experience a more painful, debilitating, and accelerated form of knee osteoarthritis. Accelerated knee osteoarthritis is defined as a person progressing from a normal joint on radiographs to advanced-stage radiographic disease within 4 years, and oftentimes this progression occurs within a single year [2, 3]. People prior to the radiographic development of accelerated knee osteoarthritis are more likely to report frequent knee pain and present with decreased physical function (e.g., slower walking and chair-stand pace) compared to those who develop a typical, gradual onset of knee osteoarthritis [2]. Additionally, people that develop accelerated knee osteoarthritis are more likely to receive pharmacological treatments and knee replacements than people who develop typical knee osteoarthritis [4, 5]. Therefore, developing prognostic methods to detect early manifestations of the disease will be key to identifying who is at high-risk for the radiographic development of accelerated knee osteoarthritis.

The development of accelerated knee osteoarthritis is characterized by extensive pre-radiographic structural pathology detected with magnetic resonance (MR) imaging when compared to people that experience the typical, gradual onset of knee osteoarthritis [6]. Greater increases in effusion-synovitis and bone marrow lesion (BML) volume [7, 8], as well as a more rapid decline in articular cartilage thickness are structural alterations observed in people prior to x-ray evidence of accelerated knee osteoarthritis compared to typical knee osteoarthritis development [8, 9]. However, traditional quantitative MR-based assessments of individual structural pathologies fail to help classify people who will develop accelerated knee osteoarthritis better than models with only clinical measures [10]. Novel approaches that combine the structural burden across multiple knee regions and pathologies may efficiently quantify total structural burden of knee osteoarthritis to account for the multifactorial etiology of the disease and help identify people at risk for accelerated knee osteoarthritis development.

We recently deployed an extensive, iterative process to create and validate a “whole-knee” MR-based composite metric based on cartilage damage, bone marrow lesions (BMLs), and effusion-synovitis [11]. Out of 12 candidate composite metrics, two emerged as superior to the others. These novel composite metrics conceptualize knee osteoarthritis progression as two constructs. First, the *disease activity* metric combines multiple regions of BML volume and effusion-synovitis volume that reflects the transient fluctuations of knee osteoarthritis pathology and is related to knee pain. Second, the *cumulative damage* metric combines cartilage damage throughout the entire tibiofemoral joint and represents the accumulation of joint damage throughout the course of disease [11]. These two composite metrics offer a novel way to conceptualize and define knee osteoarthritis progression that is consistent with frameworks used in other diseases (e.g., systemic lupus erythematosus, rheumatoid arthritis) [12, 13].

It is unclear how these composite metrics differ prior to and following the radiographic onset of accelerated knee osteoarthritis when compared to people that develop typical knee osteoarthritis or remain with no knee osteoarthritis. Additionally, it is unclear if longitudinal change in these composite metrics prior to the radiographic onset of disease is prognostic of future development of accelerated knee osteoarthritis. Therefore, our first aim was to determine if disease activity or cumulative damage differed between an accelerated or typical knee osteoarthritis group prior to and following radiographic disease development, as well as compared to a no knee osteoarthritis group over the same period. Our second aim was to determine if one-year change in disease activity or cumulative damage prior to the development of disease was associated with the future development of accelerated knee osteoarthritis. We hypothesize that there will be greater disease activity and cumulative damage prior to the onset of accelerated knee osteoarthritis, which will persist up to 2 years after disease onset. Additionally, the people exhibiting greater worsening of disease activity or cumulative damage over a one-year period before radiographic disease onset will be more likely to develop accelerated knee osteoarthritis.

## Methods

### Study design

We conducted a secondary longitudinal analysis on a nested case-control study that was designed to characterize people that developed either accelerated or typical knee osteoarthritis or remained without knee osteoarthritis within the initial 4 years of the Osteoarthritis Initiative (OAI). Within the OAI, 4796 people with or at risk for symptomatic knee osteoarthritis were enrolled at four clinical sites from February 2004 to May 2006: Memorial Hospital of Rhode Island, Ohio State University, University of Pittsburgh, and University of Maryland and Johns Hopkins [14]. Each of the OAI clinical sites, as well as the coordinating center at the University of California, San Francisco received approval from their respective institutional review boards. Prior to enrollment in the OAI, all participants provided informed consent.

### Participant selection

**Fig. 1:**
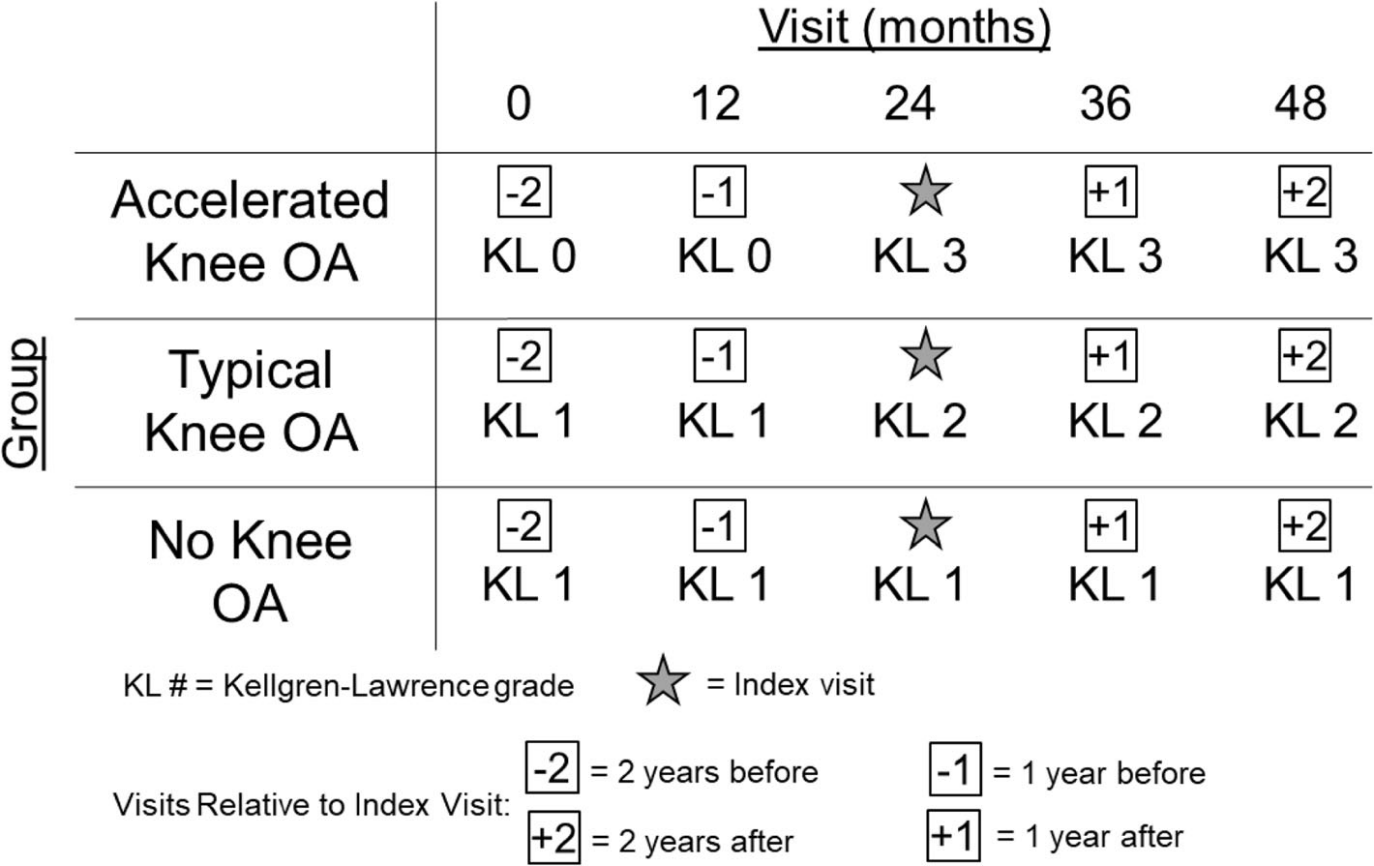
Examples of Radiographic Changes that Define the Knee Osteoarthritis Groups and the Study Observation Period. This figure provides examples of Kellgren-Lawrence (KL) grades overtime in each of the knee OA groups. We focused on visits up to 2 years prior to and up to 2 years after the index visit

We focused on people with no evidence of radiographic knee osteoarthritis (i.e., Kellgren-Lawrence [KL] grade 0/1) at the OAI baseline visit. We used the bilateral KL grades from the central OAI readers who scored the weight-bearing, fixed-flexion posteroanterior knee radiographs [3]. The knee radiographs were monitored at each visit during the initial 4 years of the OAI to define disease progression (files: kXR_SQ_BU##_SAS [versions 0.6, 1.6, 3.5, 5.5, and 6.3]) [14]. We identified three groups based on the amount of disease progression during the initial 4 years of the OAI: 1) *accelerated knee osteoarthritis*: progressed to KL 3/4 in 4 years (*n* = 125); 2) *typical knee osteoarthritis*: any other KL grade increase in 4 years (*n* = 187); 3) *no knee osteoarthritis*: KL grade remained the same over 4 years (*n* = 1325) [3]. Figure 1 provides examples of the radiographic disease progression used to define each knee osteoarthritis group. People in the typical and no knee osteoarthritis groups were matched by sex to people in the accelerated knee osteoarthritis group (i.e., 125 people per group). Figure 1 provides an example of the radiographic definition that defines group enrollment.

### Defining the index knee

The *index knee* in the accelerated knee osteoarthritis group was defined as the first knee that progressed to KL3/4 in 4 years. The *index knee* in the typical knee osteoarthritis group was defined as the first knee to increase in KL grade in 4 years. Since neither knee progressed in the no knee osteoarthritis group remained the same over 4 years, the *index knee* in this group was matched to the index limb of their matched participant in the accelerated knee osteoarthritis group.

### Defining the index visit and study observation period

The *index visit* in the accelerated and typical knee osteoarthritis groups was defined as the visit in which the person’s *index knee* met the accelerated or typical knee osteoarthritis definition, respectively. The *index visit* in the no knee osteoarthritis group was matched to the *index visit* of their matched participant in the accelerated knee osteoarthritis group. Figure 1 provides a graphical representation of how the *index visit* was defined for each group. For this study, we focused on the yearly OAI visits up to 2 years before and up to 2 years after a person’s *index visit* (Fig. 1).

### MR imaging methodology

#### MR acquisition

Each OAI site used identical Siemens Trio 3-Tesla MR systems and followed the specifications in the OAI Imaging Protocol [15]. Cartilage damage index (CDI) was assessed using a 3-dimensional dual-echo steady-state sequence. A sagittal intermediate-weighted, turbo spin echo, fat-suppressed MR sequence was used to assess effusion-synovitis bone marrow lesion and volume. These sequences have been described in detail elsewhere [15].

#### Quantifying cartilage damage index (CDI)

**Fig. 2:**
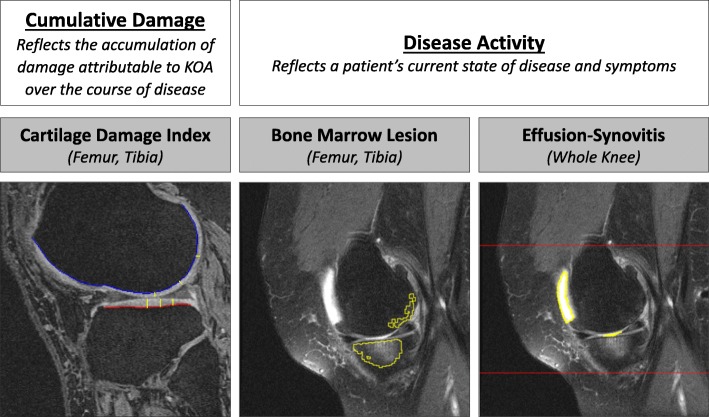
Composite Structural Metrics that Conceptualize Knee Osteoarthritis Progression as the Combination of Cumulative Damage and Disease Activity. Parsimonious semi-automated programs were used to individually assess cartilage damage index, bone marrow lesion volume, and effusion-synovitis. Cumulative damage is the sum of standardized cartilage damage (four regions: medial/lateral tibia/femur). Disease activity is the sum of the standardized volumes of effusion-synovitis (single volumetric measure) and BML (four regions: medial/lateral tibia/femur)

A single reader (JED) used a custom semi-automated program to quantify tibiofemoral CDI, a valid measure of cartilage thickness (Fig. 2) [16, 17]. Briefly, the reader would measure cartilage thickness at 36 informative locations in the medial/lateral femur/tibia (i.e., 9 each region) that were automatically located by the semi-automated program based on the width of the knee. These 36 locations were considered informative because this is where people with knee osteoarthritis are most likely to develop cartilage defects [17]. The semi-automated program produces a CDI value for all four tibiofemoral regions (medial/lateral femur/tibia). The study principal investigator (JBD) reviewed all CDI measurements and has exhibited excellent intra-reader reliability with this technique (ICC_3,1_ = 0.86 to 0.99).

#### Quantifying BML volume

A single reader (ACS) used a semi-automated program to assess BML volume (Fig. 2) [18, 19]. Based on crude bone boundaries identified by the reader, the program automatically identified the precise bone boundaries and segmented areas of high signal intensity (e.g., BML). To rule out areas of high signal intensity that were not considered a BML, we defined a BML as: 1) originating < 10 mm from the articular surface [20]; 2) needing to be present on more than one MR slice. BML volume was quantified within all four tibiofemoral regions (medial/lateral femur/tibia). The study principal investigator reviewed all BML volume measurements has exhibited excellent intra-reader reliability (ICC_3,1_ = 0.91).

#### Quantifying effusion-Synovitis volume

A single reader (JBD) used a semi-automatic program to measure whole joint knee effusion-synovitis (Fig. 2) [6, 21]. After the reader identified the proximal patellar border and the fibular head apex on a MR slice in the middle of the joint, the software automatically segmented effusion-synovitis within these manual set boundaries. After manually excluding areas of high intensity not considered effusion-synovitis, the semi-automatic program determined the effusion-synovitis volume throughout the entire knee joint. The study principal investigator reviewed all effusion-synovitis volume measurements and has exhibited demonstrated excellent intra-reader reliability (ICC_3,1_ = 0.96).

#### Normalizing individual MR measures used in composite metrics

To calculate the composite MR metrics, we used 9 MR-based measures: CDI (4 regions = medial/lateral femur/tibia), BML volume (4 regions = medial/lateral femur/tibia), and effusion-synovitis volume (1 region = whole joint). Prior to combining the individual MR measures into the composite metrics, each of the 9 measures was normalized to knee size (i.e., multiplying each measure to the ratio of the person’s bone width to the average bone width of 500 knees previously measured within the OAI). Next, we standardized the normalized measures with the means and standard deviations of the entire group at the earliest study time point (i.e., 2 years prior to the index visit): (individual normalized measure – whole group mean of normalized measure)/whole group standard deviation of normalized measure. This standardization approach has two purposes: 1) puts all MR-based measure on the same scale, 2) enhances the interpretability of the measures.

#### Creating composite metrics of MR-based knee structure

Since the MR-based measures are all on the same standardized scale, we are able to combine the measures to calculate the composite metrics: 1) *disease activity*: sum of the standardized BML volume for all four tibiofemoral regions plus the whole-joint effusion-synovitis volume; 2) *cumulative damage*: sum of the standardized CDI for all four tibiofemoral regions. To ensure that a larger value for disease activity and cumulative damage indicated worse knee pathology, we multiplied the cumulative damage metric by − 1. Additionally, interpretability of the composite metrics is improved as both disease activity and cumulative damage are interpreted as the number of standard deviations away from the overall group mean at the earliest stage of disease.

### Clinical data

Body mass index (BMI), age, race, days with limited activity in the prior month, frequent knee pain within the past month (i.e., yes or no), overall global rating, pain medication (i.e., yes or no), and Western Ontario and McMaster Universities Osteoarthritis Index (WOMAC) pain were acquired at each OAI visit. The data are publicly available (Files: allclinical0#; version 0.2.2, 1.2.1, 3.2.1, 5.2.1, 6.2.1) [14].

### Statistical analysis

#### Longitudinal differences in cumulative damage and disease activity

To determine between group differences at all time points for disease activity and cumulative damage, we used 2 generalized linear mixed models with group (3 levels) and time (up to 5 levels) as independent variables. In the presence of a significant group-by-time interaction, we used three post hoc comparisons at each time point to determine which groups differed at each individual time point. We adjusted for sex (i.e., matching variable) and factors related to missing MR data at the next visit (i.e., age, BMI, injury, frequent knee pain, days with limited activity in prior month, overall global rating, and WOMAC pain). We assessed model diagnostics on the cumulative damage and disease activity analyses and reran the analyses excluding people with potentially influential data (i.e., large Cook’s D value or Cook’s D covariance parameters). We conducted a sensitivity analysis that only included individuals with KL 0 at the OAI baseline visit (accelerated, *n* = 42; typical, *n* = 71; no knee osteoarthritis, *n* = 92; no matching).

#### Association between one-year change in composite metrics and future onset of accelerated knee osteoarthritis

We calculated a one-year change in cumulative damage and disease activity from two to 1 year prior to the index visit (i.e., during a pre-radiographic phase of disease). We used two multinomial logistic regressions models to determine if a one-year change in disease activity or cumulative damage was associated with the future development of accelerated knee osteoarthritis compared to the development of typical or no knee osteoarthritis. Odds ratios are reported as increased odds for developing accelerated knee osteoarthritis per standard deviation for one-year change in composite metric. Using covariates from a similar prognostic osteoarthritis imaging analysis, we adjusted for sex, race, and baseline age, BMI, KL grade, WOMAC pain, and use of pain medication [22].

## Results

### Longitudinal differences in cumulative damage and disease activity

**Fig. 3:**
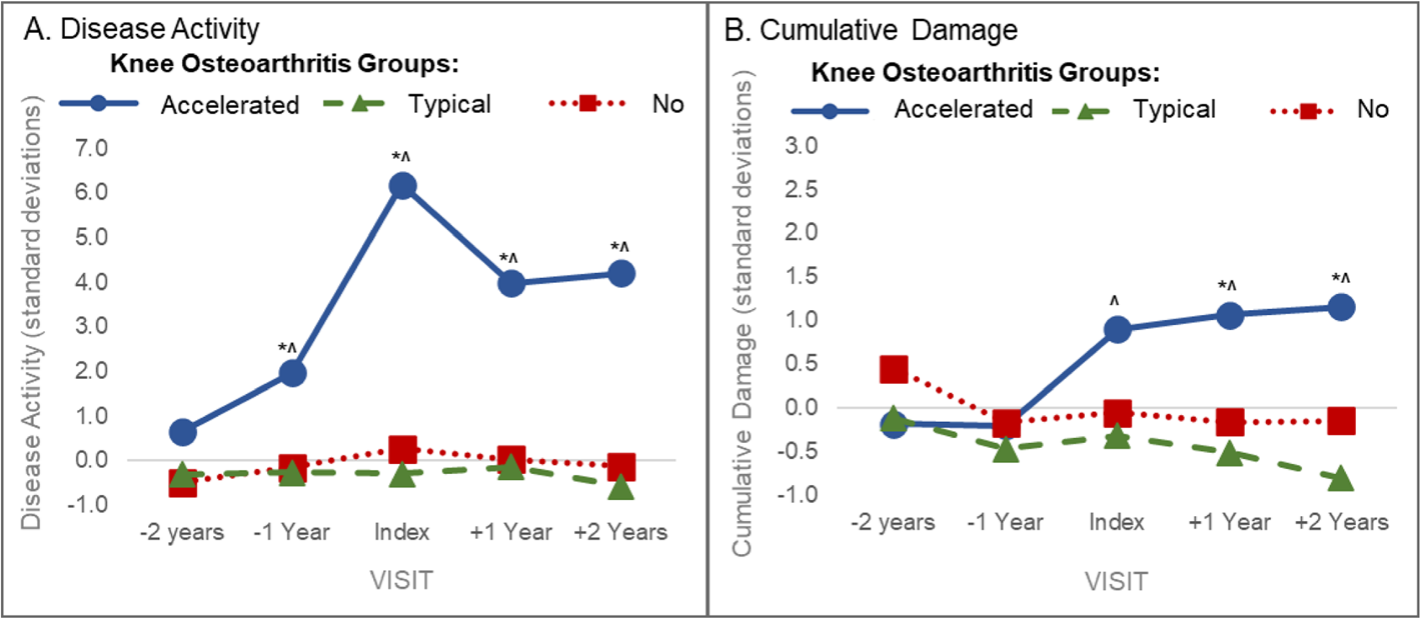
Differences in Composite Structural Metrics in Individuals with Accelerated Compared to Typical and No Knee Osteoarthritis. A. Disease Activity. B. Cumulative Damage. Statistically siginificant differences in accelerated compared to typical(*) or no (^) knee osteoarthritis. Adjusted for sex and factors related to missing magnetic resonance data at the next visit (i.e., age, body mass index, injury, frequent knee pain, days with limited activity in prior month, overall global rating, and WOMAC pain)

Table 1 provides the baseline characteristics for each group and demographic comparisons between the accelerated, typical, and no knee osteoarthritis groups. Figure 3 depicts the disease activity and cumulative damage over time in people with accelerated, typical, or no knee osteoarthritis. Starting at 1 year prior and continuing to at least 2 years after the index visit, the accelerated knee osteoarthritis group presented with greater disease activity compared to the typical and no knee osteoarthritis groups (Fig. 3). At the index visit, the mean disease activity for people that develop accelerated knee osteoarthritis is 6.1 standard deviations greater than the overall group mean compared with the other two groups that are only 0.1 (typical knee osteoarthritis) and − 0.3 (no knee osteoarthritis) standard deviations away from the overall group mean.

**Table 1:** Group Baseline Characteristics

	Knee Osteoarthritis Group			
Variables	Accelerated	Typical	No	
[(means (SD); except where noted)	(n = 125)	(n = 125)	(n = 125)	P*
Females, n(%)	79 (63%)	79 (63%)	79 (63%)	1.00
Age (years)	62.5 (8.5)*	58.4 (8.4)	57.3 (8.2)	< 0.001
Body mass index (kg/m^2^)	29.7 (4.6)*	28.1 (4.4)	26.9 (4.4)	< 0.001
Index knee KL Grade = 0, n(%)	42 (34%)*	71 (57%)^	92 (74%)	< 0.001
WOMAC pain (0–20; ↑score = ↑pain)	2.6 (3.3)	1.6 (2.1)	1.4 (2.0)	0.08
Overall Global Rating (0–10, ↑score = ↑effect)	1.7 (1.9)*	1.1 (1.5)	0.8 (1.1)	< 0.001
Days with limited activity in prior month	3.2 (7.3)^	1.7 (4.8)	1.4 (4.3)	0.03

*P* values are for the overall group main effect from a one-way analysis of variance (for continuous measures) or chi-square tests (for dichotomous variables). * = *P* < 0.017 versus typical knee osteoarthritis and no knee osteoarthritis, ^ = *P* < 0.017 versus no knee osteoarthritis

Starting after the index visit, the accelerated knee osteoarthritis group had worse cumulative damage compared to the typical and no knee osteoarthritis groups (Fig. 3). The cumulative damage in the people with accelerated knee osteoarthritis increases with each successive year, with the greatest cumulative damage occurring at 2 years after the index visit (i.e., mean cumulative damage = 1.5 standard deviations).

The typical and no knee osteoarthritis groups were not statistically significantly different at any time points for disease activity and cumulative damage (Fig. 3). Omitting influential data (e.g., based on a large Cook’s D) did not change the patterns of longitudinal alterations in disease activity and cumulative damage. In our sensitivity analysis that only included individuals with KL 0 at OAI baseline, we confirmed the statistically significant results in the primary analysis.

### Association between one-year change in composite metrics and future onset of accelerated knee osteoarthritis

Table 2 demonstrates the means and standard deviations of the one-year change in disease activity and cumulative damage from two to one years prior to the index visit for participants that develop accelerated, typical, or no knee osteoarthritis. Greater worsening of disease activity and cumulative damage from two to one years prior to the index visit is associated with the development of accelerated knee osteoarthritis compared to both typical and no knee osteoarthritis development. Specifically, for every standard deviation of disease activity worsening there is a 58 and 139% increase in the odds of developing accelerated knee osteoarthritis compared to typical and no knee osteoarthritis, respectively (Table 2). For every standard deviation of cumulative damage worsening there is a 69 and 111% increase in the odds of developing accelerated knee osteoarthritis compared to typical and no knee osteoarthritis, respectively (Table 2). In our sensitivity analysis that only included individuals with KL 0 at OAI baseline, we confirmed the statistically significant results in the primary analysis.

**Table 2:** Longitudinal Change in Cumulative Damage and Disease Activity From Two to One Year Prior to the Index Visit is Associated with Accelerated Knee Osteoarthritis (AKOA) Development

	Knee Osteoarthritis (KOA) Group			OR (95% Confidence Interval)^**a**^ OR per unit of SD^**b**^	
1-Year Change in Composite Metrics	Accelerated (***n*** = 87) Mean (SD)	Typical (***n*** = 64) Mean (SD)	No (***n*** = 96) Mean (SD)	Accelerated vs Typical KOA (REF)	Accelerated vs No KOA (REF)
Cumulative Damage	0.34 (0.70)	0.10 (0.44)	0.01 (0.55)	1.69 (1.14–2.51)^	2.11 (1.41–3.16)^
Disease Activity	1.49 (3.20)	0.61 (1.72)	0.06 (1.52)	1.58 (1.08–2.33)^	2.39 (1.55–3.71)^

*OR* odds ratio,

^**a**^Adjusted for age, BMI, sex, race, WOMAC pain, KL grade, and pain medication at the visit 2 years prior to the index visit

^**b**^ Odds ratios are reported as increased odds for developing accelerated knee osteoarthritis per entire group standard deviation for one-year change in cumulative damage (i.e., 0.60) and disease activity (i.e., 2.37)

^Statistically significant (*p*< 0.05)

## Discussion

Composite structural metrics of disease activity and cumulative damage offer a parsimonious and novel strategy to conceptualize the onset and progression of knee osteoarthritis, especially accelerated knee osteoarthritis. Disease activity, which is a dynamic metric of structural changes, is greater in people that develop accelerated knee osteoarthritis starting at 1 year prior to radiographic onset and up to at least 2 years following their index visit. Additionally, at all visits after the index visit, cumulative damage is greater in people that developed accelerated knee osteoarthritis compared to typical or no knee osteoarthritis. Prior to the onset of disease, an annual change of both cumulative damage and disease activity were associated with future radiographic development of accelerated knee osteoarthritis compared to typical and no knee osteoarthritis development. These results agree with previous reports that people who develop accelerated knee osteoarthritis present with a different pattern of pre-radiographic structural changes compared to people that develop typical knee osteoarthritis [6–9, 23]. Conceptualizing accelerated knee osteoarthritis development as two different constructs (i.e., cumulative damage and disease activity) using quantitative composite metrics may provide a robust way to structurally define the onset and development of this debilitating subset of knee osteoarthritis.

We intentionally used widely available imaging techniques and relatively simple analyses to create quantitative composite knee structure metrics to overcome some of the technical challenges of traditional quantitative and semi-quantitative knee osteoarthritis structural assessments. First, traditional quantitative knee osteoarthritis structural assessments tend to primarily focus on articular cartilage and often require 2 to 6 h per knee to segment cartilage [24, 25]. In contrast, our composite metrics combine cartilage damage with BML and effusion-synovitis volume to conceptualize knee osteoarthritis as a disease of multi-tissue failure [26]. Additionally, the individual components of the composite metrics are assessed using time-efficient techniques that need minimal researcher effort and can process the entire knee within minutes. Second, while current semi-quantitative scoring systems provide an assessment of whole joint disease, these systems yield over 100 ordinal or dichotomous scores that cannot be combined into composite scores [27]. A Rasch analysis of a common semi-quantitative knee osteoarthritis scoring system “raised important questions about summating ordinal scores from multiple anatomical sites” because it “does not always produce a valid summed score for a unidimensional scale” [28]. Thus, these composite structural metrics of cumulative damage and disease activity are: 1) time-efficient, 2) offer a whole knee approach to define disease progression, and 3) improve interpretability of whole-joint disease burden.

Disease activity is a composite of the extent of BMLs and effusion-synovitis throughout the joint, which are both independently associated with joint symptoms [11, 19, 29–31]. At a year prior to the development of accelerated knee osteoarthritis, people present with elevated disease activity when compared to people that will eventually develop typical knee osteoarthritis or remain with no knee osteoarthritis. This elevation in disease activity peaks at the index visit and persists for at least 2 years after the onset of disease (Fig. 3). This elevation in disease activity is consistent with prior findings of greater pre-radiographic pain and functional limitations in people that go on to develop accelerated knee osteoarthritis compared to those that develop typical knee osteoarthritis [2]. These findings agree with previous reports that separately report greater BML and effusion-synovitis volume in people prior to accelerated knee osteoarthritis development [7, 8]. However, since people likely experience both BMLs and effusion-synovitis, a composite metric that combines these structural features provides a better estimation of the whole-joint disease burden that defines a patient’s current state of disease and symptoms compared to each individual feature.

Cumulative damage reflects the accumulation of damage attributable to knee osteoarthritis over the course of the disease and relates to radiographic knee osteoarthritis severity. Our findings provide construct validity of the cumulative damage metric because cumulative damage fails to differentiate accelerated and typical knee osteoarthritis until after the index visit, which is when radiographic changes to joint space occur. However, an annual change in cumulative damage over a pre-radiographic disease period is associated with the future onset of accelerated disease. Specifically, for every standard deviation of cumulative damage worsening prior to disease development participants were at a 69 and 111% chance of developing accelerated knee osteoarthritis compared to typical and no knee osteoarthritis, respectively. This highlights that people with accelerated knee osteoarthritis are accumulating damage faster than people with typical knee osteoarthritis even during this pre-radiographic phase when x-rays are too insensitive to monitor disease progression. The cumulative damage metric offers a parsimonious strategy to conceptualize cartilage damage throughout the joint rather than relying on at least four different regional metrics. While these results provide an overall estimation of cumulative damage throughout the tibiofemoral joint, previous reports have demonstrated that starting at the index visit the people with accelerated knee osteoarthritis had greater cartilage damage in the medial and lateral tibia as well as the medial femur than those with typical or no knee osteoarthritis [8]. Additionally, people that develop accelerated knee osteoarthritis present with a more diffuse spatial pattern of cartilage change throughout the tibiofemoral compartment compared to those that develop typical knee osteoarthritis [9]. Hence, an advantage to using cumulative damage in analyses is that it efficiently describes the damage associated with accelerated knee osteoarthritis without the need for numerous analyses.

While this study highlights the utility of using composite metrics to define the burden and progression of structural disease prior to the development of accelerated knee osteoarthritis, there are some limitations that need to be mentioned. Our composite metrics use a combination of whole joint articular cartilage damage, BML volume, and effusion-synovitis volume to quantify metrics of cumulative damage and disease activity; however, there are other joint structures that play a role in the onset and progression of knee osteoarthritis. Additionally, our structural metrics focused solely on the tibiofemoral joint and we acknowledge that not including the patellofemoral compartment is a limitation. Future studies are needed to develop parsimonious, quantitative methods for quantifying outcomes that assess other relevant joint structures (e.g., meniscus, tendons, and ligaments) that can be incorporated into the composite structural metrics. Furthermore, future studies of accelerated knee osteoarthritis should also consider the patellofemoral compartment as 66–75% of people in all three groups present with MR evidence of patellofemoral osteoarthritis [7].The sample size of this study was relatively small since we focused on the role of these composite metrics at predicting the onset of accelerated knee osteoarthritis. Future studies are needed to utilize these techniques in a larger study to better discern how these composite metrics can be used to identify other subsets or phenotypes of knee osteoarthritis.

## Conclusions

In conclusion, composite knee osteoarthritis metrics that conceptualize structural disease progression as cumulative damage or disease activity are elevated in people prior to and following the onset and progression of accelerated knee osteoarthritis. Additionally, an annual change in both composite metrics during a pre-radiographic stage of disease predicts the accelerated onset of disease. These composite metrics of knee osteoarthritis structural damage offer a more time-efficient interpretable method of quantifying whole-joint disease burden when compared to traditional techniques.

## Data Availability

The datasets generated and/or analyzed during the current study are available in the OAI repository, https://nda.nih.gov/oai/

## Abbreviations

MR: Magnetic resonance
KL: Kellgren-Lawrence
BML: Bone marrow lesion
OAI: Osteoarthritis Initiative
CDI: Cartilage damage index
ICC: Intraclass correlation coefficient
BMI: Body mass index
WOMAC: Western Ontario and McMaster Universities Osteoarthritis Index

## References

[1] Cross M, Smith E, Hoy D, Nolte S, Ackerman I, Fransen M, Bridgett L, Williams S, Guillemin F, Hill CL (2014). The global burden of hip and knee osteoarthritis: estimates from the global burden of disease 2010 study. Ann Rheum Dis.

[2] Driban JB, Price LL, Eaton CB, Lu B, Lo GH, Lapane KL, McAlindon TE (2016). Individuals with incident accelerated knee osteoarthritis have greater pain than those with common knee osteoarthritis progression: data from the osteoarthritis initiative. Clin Rheumatol.

[3] Driban JB, Stout AC, Lo GH, Eaton CB, Price LL, Lu B, Barbe MF, McAlindon TE (2016). Best performing definition of accelerated knee osteoarthritis: data from the osteoarthritis initiative. Ther Adv Musculoskelet Dis.

[4] Davis JE, Liu SH, Lapane K, Harkey MS, Price LL, Lu B, Lo GH, Eaton CB, Barbe MF, McAlindon TE (2018). Adults with incident accelerated knee osteoarthritis are more likely to receive a knee replacement: data from the osteoarthritis initiative. Clin Rheumatol.

[5] Davis JE, Harkey MS, Liu SH, Lapane K, Price LL, Lu B, Lo GH, Eaton CB, Barbe MF, McAlindon TE (2019). Adults with incident accelerated knee osteoarthritis are more likely to use pharmacological treatment options and receive arthroscopic knee surgery: data from the osteoarthritis initiative. ACR Open Rheumatol.

[6] Harkey MS, Davis JE, Lu B, Price LL, Ward RJ, MacKay JW, Eaton CB, Lo GH, Barbe MF, Zhang M (2019). Early pre-radiographic structural pathology precedes the onset of accelerated knee osteoarthritis. BMC Musculoskelet Disord.

[7] Davis JE, Ward RJ, MacKay JW, Lu B, Price L, McAlindon TE, Eaton CB, Barbe MF, Lo GH, Harkey MS (2018). Effusion-synovitis and infrapatellar fat pad signal intensity alteration differentiate accelerated knee osteoarthritis. Rheumatology.

[8] Driban JB, Davis JE, Lu B, Price L, Ward RJ, MacKay JW, Eaton CB, Lo GH, Barbe MF, Zhang M (2019). Accelerated knee osteoarthritis is characterized by destabilizing meniscal tears and Preradiographic structural disease burden. Arthritis Rheumatol.

[9] Harkey MS, Davis JE, Lu B, Price LL, Eaton CB, Lo GH, Barbe MF, Ward RJ, Zhang M, Liu SH (2019). Diffuse tibiofemoral cartilage change prior to the development of accelerated knee osteoarthritis: data from the osteoarthritis initiative. Clin Anat.

[10] Price LL, Harkey MS, Ward RJ, MacKay JW, Zhang M, Pang J, Davis JE, McAlindon TE, Lo GH, Amin M (2019). Role of magnetic resonance imaging in classifying individuals who will develop accelerated radiographic knee osteoarthritis. J Orthop Res.

[11] Price LL, Driban JB, Lo GH, Zhang M, LaValley MP, McAlindon TE (2018). A new way to think about composite magnetic resonance imaging scores to measure osteoarthritis severity and progression. Arthritis Rheumatol.

[12] Li W, Sasso EH, AHMvdH-v M, TWJ H (2015). Relationship of multi-biomarker disease activity score and other risk factors with radiographic progression in an observational study of patients with rheumatoid arthritis. Rheumatol Oxf Engl.

[13] Fortin PR, Abrahamowicz M, Neville C, Berger RD, Fraenkel L, Clarke AE, Danoff D (1998). Impact of disease activity and cumulative damage on the health of lupus patients. Lupus.

[14] Eckstein F, Wirth W, Nevitt MC (2012). Recent advances in osteoarthritis imaging--the osteoarthritis initiative. Nat Rev Rheumatol.

[15] Peterfy CG, Schneider E, Nevitt M (2008). The osteoarthritis initiative: report on the design rationale for the magnetic resonance imaging protocol for the knee. Osteoarthr Cartil.

[16] Zhang M, Driban JB, Price LL, Lo GH, Miller E, McAlindon TE (2015). Development of a rapid cartilage damage quantification method for the lateral Tibiofemoral compartment using magnetic resonance images: data from the osteoarthritis initiative. Biomed Res Int.

[17] Zhang M, Driban JB, Price LL, Harper D, Lo GH, Miller E, Ward RJ, McAlindon TE (2014). Development of a rapid knee cartilage damage quantification method using magnetic resonance images. BMC Musculoskelet Disord.

[18] Pang J, Driban JB, Destenaves G, Miller E, Lo GH, Ward RJ, Price LL, Lynch JA, Eaton CB, Eckstein F (2013). Quantification of bone marrow lesion volume and volume change using semi-automated segmentation: data from the osteoarthritis initiative. BMC Musculoskelet Disord.

[19] Driban JB, Price L, Lo GH, Pang J, Hunter DJ, Miller E, Ward RJ, Eaton CB, Lynch JA, McAlindon TE (2013). Evaluation of bone marrow lesion volume as a knee osteoarthritis biomarker--longitudinal relationships with pain and structural changes: data from the osteoarthritis initiative. Arthritis Res Ther.

[20] Roemer FW, Frobell R, Hunter DJ, Crema MD, Fischer W, Bohndorf K, Guermazi A (2009). MRI-detected subchondral bone marrow signal alterations of the knee joint: terminology, imaging appearance, relevance and radiological differential diagnosis. Osteoarthr Cartil.

[21] Davis JE, Ward RJ, MacKay JW, Lu B, Price LL, McAlindon TE, Eaton CB, Barbe MF, Lo GH, Harkey MS, et al. Effusion-synovitis and infrapatellar fat pad signal intensity alteration differentiate accelerated knee osteoarthritis. Rheumatology (Oxford). 2018:58(3):418–26. 10.1093/rheumatology/key305 PMC6381765 30346594

[22] Collins JE, Losina E, Nevitt MC, Roemer FW, Guermazi A, Lynch JA, Katz JN, Kent Kwoh C, Kraus VB, Hunter DJ (2016). Semiquantitative imaging biomarkers of knee osteoarthritis progression: data from the Foundation for the National Institutes of Health osteoarthritis biomarkers consortium. Arthritis Rheumatol.

[23] Davis JE, Harkey MS, Ward RJ, MacKay JW, Lu B, Price LL, Eaton CB, Lo GH, Barbe MF, McAlindon TE (2019). Accelerated knee osteoarthritis is associated with pre-radiographic degeneration of the extensor mechanism and cruciate ligaments: data from the osteoarthritis initiative. BMC Musculoskelet Disord.

[24] Shim H, Chang S, Tao C, Wang JH, Kwoh CK, Bae KT (2009). Knee cartilage: efficient and reproducible segmentation on high-spatial-resolution MR images with the semiautomated graph-cut algorithm method. Radiology.

[25] Eckstein F, Wirth W (2011). Quantitative cartilage imaging in knee osteoarthritis. Arthritis.

[26] Loeser RF, Goldring SR, Scanzello CR, Goldring MB (2012). Osteoarthritis: a disease of the joint as an organ. Arthritis Rheum.

[27] Hunter DJ, Guermazi A, Lo GH, Grainger AJ, Conaghan PG, Boudreau RM, Roemer FW (2011). Evolution of semi-quantitative whole joint assessment of knee OA: MOAKS (MRI Osteoarthritis Knee Score). Osteoarthritis Cartilage.

[28] Conaghan PG, Tennant A, Peterfy CG, Woodworth T, Stevens R, Guermazi A, Genant H, Felson DT, Hunter D (2006). Examining a whole-organ magnetic resonance imaging scoring system for osteoarthritis of the knee using Rasch analysis. Osteoarthritis Cartilage.

[29] Hunter DJ, Zhang W, Conaghan PG, Hirko K, Menashe L, Li L, Reichmann WM, Losina E (2011). Systematic review of the concurrent and predictive validity of MRI biomarkers in OA. Osteoarthr Cartil.

[30] Lo GH, McAlindon TE, Niu J, Zhang Y, Beals C, Dabrowski C, Le Graverand MP, Hunter DJ, Group OAII (2009). Bone marrow lesions and joint effusion are strongly and independently associated with weight-bearing pain in knee osteoarthritis: data from the osteoarthritis initiative. Osteoarthr Cartil.

[31] Felson DT, Chaisson CE, Hill CL, Totterman SM, Gale ME, Skinner KM, Kazis L, Gale DR (2001). The association of bone marrow lesions with pain in knee osteoarthritis. Ann Intern Med.

